# The CALLY index, a novel composite biomarker, is inversely associated with preserved ratio impaired spirometry (PRISm): A nationally representative cross-sectional study

**DOI:** 10.1097/MD.0000000000051472

**Published:** 2026-11-06

**Authors:** Yucai Ye, Youhong Zhao, Liya Jin, Juanxue Zhang, Qiong Jia, Wenjuan Bai, Yanjiong Wang

**Affiliations:** a Health Management Center, Gansu Province Central Hospital, Lanzhou, P.R. China.

**Keywords:** C-reactive protein-albumin-lymphocyte index, CALLY index, inflammation, NHANES, Preserved ratio impaired spirometry

## Abstract

Preserved ratio impaired spirometry (PRISm) is an intermediate pulmonary phenotype with heightened cardiometabolic risk. Effective tools for its identification are lacking. However, its relationship with systemic inflammatory-nutritional balance is underexplored. The CALLY index integrates C-reactive protein, albumin, and lymphocyte count. This composite biomarker reflects inflammatory-nutritional homeostasis. This study aimed to investigate the association between the ln(CALLY) index and PRISm in a nationally representative U.S. adult population. We analyzed data from 7296 participants in the National Health and Nutrition Examination Survey (NHANES) 2007–2010. PRISm was defined as FEV1/FVC ≥ 0.7 with FEV1 < 80% predicted. Multivariable logistic regression models assessed the association between ln(CALLY) (as quartiles and per-standard-deviation increases) and PRISm, with sequential adjustment for demographic, socioeconomic, and cardiometabolic factors. Restricted cubic splines examined the dose-response relationship. Subgroup and sensitivity analyses evaluated the robustness of findings. Among participants, 920 (12.6%) had PRISm. Compared to those with normal pulmonary function, PRISm individuals had significantly lower ln(CALLY) values (1.21 vs 1.77, *P* < .001). In fully adjusted models, higher ln(CALLY) quartiles had progressively lower PRISm odds: Q2 (OR = 0.71, 95% CI:0.57–0.88), Q3 (OR = 0.63, 0.47–0.84), and Q4 (OR = 0.48, 0.35–0.66) vs Q1 (*P*-trend < 0.001). Each standard deviation increase in ln(CALLY) reduced PRISm risk by 24% (OR = 0.76, 0.68–0.85). Restricted cubic spline analysis showed a monotonic inverse linear dose-response relationship (*P*-overall < .001, *P*-nonlinear = .707). Subgroup analyses found stronger protective associations in women (*P*-interaction = .021) and significant heterogeneity across racial/ethnic groups (*P*-interaction = .036). Sensitivity analyses treating BMI continuously and using multiple imputation supported these findings. Higher ln(CALLY) is independently associated with lower PRISm prevalence in U.S. adults. Systemic inflammatory-nutritional status may be a modifiable risk factor for PRISm, supporting potential targeted prevention strategies. The CALLY index, derived from routine blood tests, could serve as a readily accessible tool for risk stratification of PRISm.

## 1. Introduction

Preserved ratio impaired spirometry (PRISm), characterized by a normal FEV1/FVC ratio (≥0.7) but a reduced FEV1(<80% of predicted), has recently emerged as a research focus in respiratory medicine as an intermediate spirometric phenotype. Epidemiological studies indicate a prevalence of approximately 5 to 15% in the general population, with significant variation across regions and populations. PRISm is strongly associated with significantly increased risks of all-cause mortality, cardiovascular disease, diabetes, and other comorbidities. Some patients may also progress to chronic obstructive pulmonary disease (COPD), underscoring the need for clinical attention to its long-term prognosis.^[1–3]^ Despite its growing clinical significance, the pathophysiological mechanisms underlying PRISm remain insufficiently understood, particularly the roles of systemic inflammation and nutritional status in its development and progression.

Systemic inflammation and immune dysregulation are important in chronic lung diseases.^[4,5]^ C-reactive protein (CRP) is a sensitive marker of systemic inflammation and a classic acute-phase reactant. Serum albumin reflects nutritional status and also has anti-inflammatory and antioxidant effects. Lymphocyte count measures the body’s adaptive immune response. The CALLY index (C-reactive protein, Albumin, Lymphocytes index) combines these three markers. It provides a comprehensive view of the body’s inflammation, nutrition, and immune balance.^[6]^ The CALLY index was first used in oncology research and has been shown to predict outcomes across various cancers.^[7–9]^ However, its use in respiratory diseases is still new. No studies have investigated its role in PRISm.

Given the intertwined nature of systemic inflammation, nutritional status, and pulmonary health, coupled with the clinical need to identify individuals with PRISm, we hypothesize that the CALLY index – an integrated measure of inflammation, nutrition, and immunity – is inversely associated with PRISm risk. Utilizing data from the nationally representative NHANES, this study aims to be the first to comprehensively investigate this association and its dose-response pattern. Our findings are anticipated to provide novel epidemiological evidence not only for understanding the systemic pathophysiology of PRISm but also for evaluating the potential of this easily obtainable biomarker for risk stratification of PRISm.

## 2. Methods

### 2.1. Study population

The data for this study were obtained from the National Health and Nutrition Examination Survey (NHANES) database, a nationally representative cross-sectional survey conducted by the Centers for Disease Control and Prevention (CDC) in collaboration with the National Center for Health Statistics (NCHS). NHANES collects demographic, health status, laboratory, and nutritional data through a complex multistage probability sampling design that includes household interviews, mobile examination centers, and biospecimen collection.^[10]^ The study protocol was approved by the NCHS Research Ethics Review Board, and informed consent was obtained from all participants. This study utilized NHANES data from the 2007 to 2010 cycles for cross-sectional analysis. The primary objective was to investigate the association between the ln(CALLY) index and PRISm.

Participants from two consecutive NHANES cycles (2007–2010) were initially included. The participant selection process is detailed in Figure 1. Inclusion criteria were: age 20 to 80 years; complete pulmonary function data; complete data on CALLY index components (C-reactive protein, albumin, lymphocyte count); and availability of data on key covariates. Exclusion criteria included: age < 20 or > 80 years; missing pulmonary function or CALLY-related data; physiologically implausible extreme values (e.g., FEV1 < 0.5L or > 8L, CRP > 10 mg/dL, BMI < 16 or > 60 kg/m^2^); and spirometric airflow obstruction (pre-bronchodilator FEV1/FVC < 0.70). The final analytical sample comprised 7296 participants (Fig. 1).

**Figure 1.**
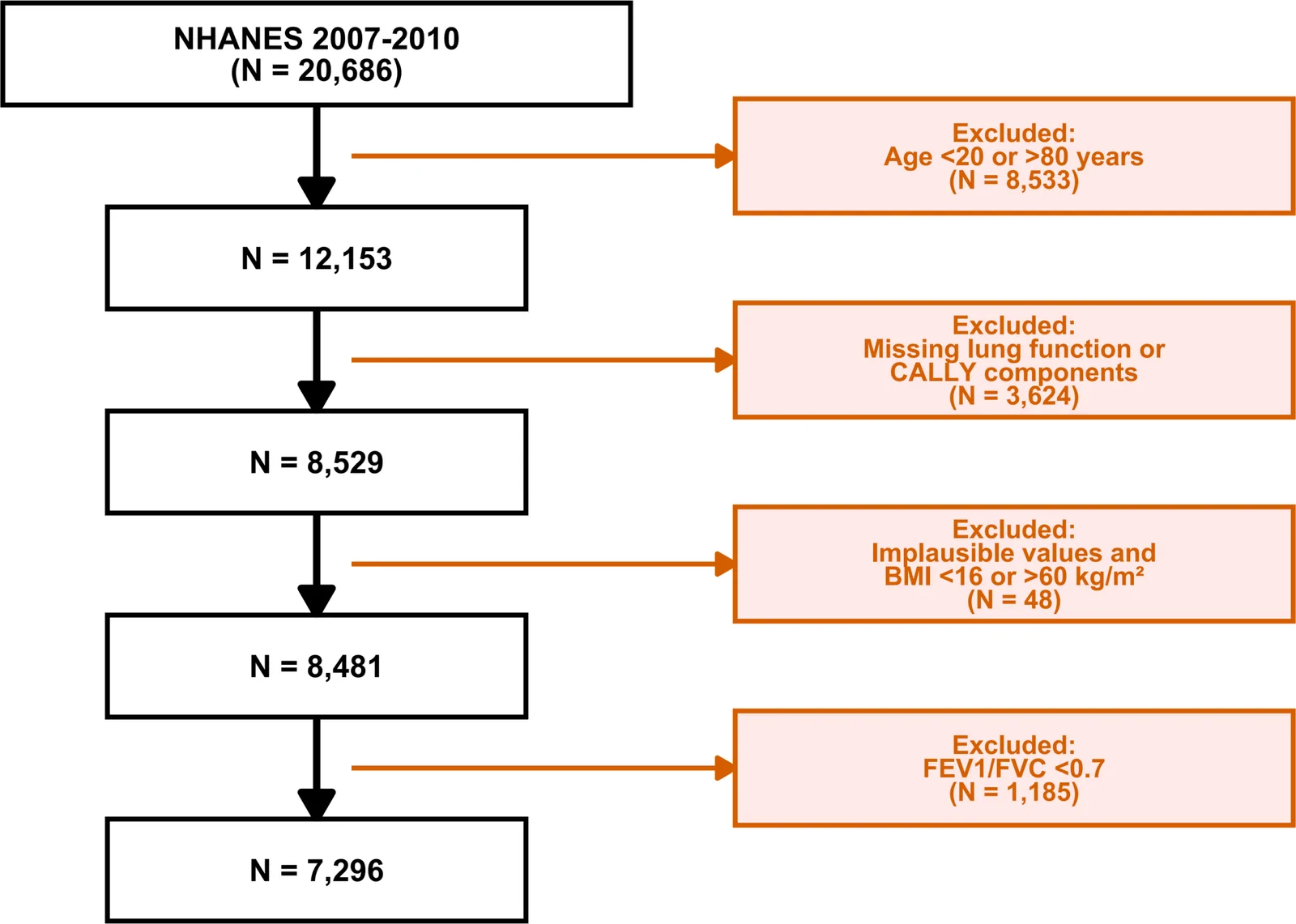
Flowchart of participant selection in the National Health and Nutrition Examination Survey (NHANES) 2007–2010 study population. Participants were excluded for age outside 20–80 yr, missing pulmonary function or CALLY index component data, physiologically implausible extreme values, and spirometric airflow obstruction (pre-bronchodilator FEV1/FVC < 0.70). A total of 7296 participants were finally included in the cross-sectional analysis.

### 2.2. Definitions of PRISm, CALLY index, and log transformation

In this study, PRISm was defined as an FEV1/FVC ratio ≥ 0.7 with FEV1 < 80% predicted. Pulmonary function data were measured by trained technicians following standardized spirometry protocols established by the American Thoracic Society/European Respiratory Society (ATS/ERS). Spirometry was performed strictly in accordance with ATS/ERS diagnostic criteria. Acceptable and reproducible maneuvers were required for valid measurements, with the highest two FEV1 and FVC values differing by ≤ 150 mL to ensure testing quality and minimize measurement bias. The FEV1 percent predicted was calculated using the NHANES III reference equations.^[11]^ The CALLY index was calculated as: CALLY = [Serum albumin (g/dL) × Lymphocyte count (×10^9^/L)]/ [C-reactive protein (mg/dL) × 10].^[12]^ Due to its skewed distribution, a natural logarithmic transformation was applied to obtain the ln(CALLY) index for primary analysis.

### 2.3. Definition of covariates

Covariates primarily included demographic and socioeconomic characteristics, as well as established PRISm risk factors. Demographic characteristics comprised age (continuous variable, also categorized as < 60 and ≥ 60 years), sex, and race/ethnicity (Non-Hispanic White, Mexican American, Other Hispanic, Non-Hispanic Black, Other Race). Socioeconomic factors included education level (<high school graduation vs ≥high school graduation), marital status (married/cohabiting vs single/separated/divorced/widowed), and family poverty-income ratio (PIR, <1.30 vs ≥1.30). Established risk factors included smoking status (never smoker vs former/current smoker), alcohol consumption (nondrinker vs drinker), body mass index (BMI, continuous variable and categorized as normal/underweight, overweight, obese), diabetes (based on self-reported physician diagnosis), and hypertension (based on self-reported physician diagnosis or current use of antihypertensive medication). All covariates were assessed through standardized NHANES interviews or physical examination questionnaires.

### 2.4. Statistical analysis

After data organization, weighted analyses were conducted to represent the noninstitutionalized civilian U.S. population. Baseline characteristics were presented stratified by PRISm status. For continuous variables, weighted means with their standard errors were calculated and compared using weighted Student’s t tests. For categorical variables, weighted percentages (accompanied by unweighted counts) were calculated and compared using Rao-Scott adjusted chi-squared tests.

Multivariable logistic regression models were employed to assess the association between the ln(CALLY) index (analyzed as quartiles and per standard deviation increase) and PRISm. Three sequential models were constructed: Model 1 (unadjusted), Model 2 (adjusted for demographic factors: age, sex, race/ethnicity), and Model 3 (additionally adjusted for socioeconomic and cardiometabolic factors: education, marital status, poverty-income ratio, smoking status, alcohol use, BMI, diabetes, hypertension). Odds ratios (ORs) with their 95% confidence intervals (CIs) were used to quantify the strength of association. The *P*-value for trend across quartiles was assessed by entering the ln(CALLY) quartiles as an ordinal variable into the model.

To explore the dose-response relationship and test for potential nonlinearity, restricted cubic splines (RCS) with four knots placed at the 5th, 35th, 65th, and 95th percentiles of the ln(CALLY) distribution were employed. nonlinearity was assessed by comparing the model with the RCS term against a model with a linear term using a likelihood ratio test (reference 6).

Pre-specified subgroup analyses and interaction tests were conducted based on the fully adjusted Model 3. Subgroups included age, sex, race/ethnicity, education level, marital status, poverty-income ratio, BMI category, smoking status, alcohol consumption, diabetes, and hypertension. Interaction was tested by including multiplicative interaction terms and evaluating their significance.

To ensure the robustness of the findings, sensitivity analyses were performed: treating BMI as a continuous variable rather than a categorical variable; and applying multiple imputation by chained equations (MICE, m = 10) for missing covariate data, with estimates combined using Rubin’s rules.As a sensitivity analysis, airflow obstruction was defined using GLI-2012 z-scores (FEV1/FVC z-score < −1.645, corresponding to the lower limit of normal) instead of the fixed ratio of 0.70. PRISm was defined as FEV1/FVC z-score ≥ −1.645 with FEV1 < 80% predicted.

All statistical analyses were performed using R software (version 4.2.1) with the survey (version 4.2–1) and rms (version 6.8–0) packages. A two-sided *P*-value < .05 was considered statistically significant.

## 3. Results

### 3.1. Baseline characteristics

The baseline characteristics of the study population stratified by PRISm status are presented in Table 1. Among 7296 participants, 920 (12.6%) had PRISm. Compared to participants with normal pulmonary function, those with PRISm were significantly older, had a higher prevalence of Non-Hispanic Black ethnicity, and exhibited a poorer cardiometabolic profile (e.g., higher BMI, and greater prevalence of diabetes and hypertension, all *P* < .001). Crucially, both the CALLY score and its log-transformed value [ln(CALLY)] were significantly lower in the PRISm group (ln(CALLY): 1.21 vs 1.77, *P* < .001), with a clear graded inverse distribution across its quartiles.

**Table 1:** Baseline characteristics of the study population (weighted).

Characteristic	Total (n = 7296)	PRISm (n = 920)	Normal pulmonary function (n = 6376)	*P*-value
**Age (years**)	43.23 ± 0.33	47.80 ± 0.75	42.75 ± 0.33	<.001
**Age group**				<.001
** **<60 yr	84.2% (5615)	76.6% (625)	85.0% (4990)	
** **≥60 yr	15.8% (1681)	23.4% (295)	15.0% (1386)	
**Gender**				.847
** **Female	52.0% (3830)	52.4% (484)	52.0% (3346)	
** **Male	48.0% (3466)	47.6% (436)	48.0% (3030)	
**Race**				<.001
** **Non-hispanic White	67.6% (3239)	42.2% (236)	70.2% (3003)	
** **Mexican American	9.7% (1510)	4.1% (75)	10.3% (1435)	
** **Other hispanic	5.6% (873)	4.5% (79)	5.7% (794)	
** **Non-hispanic Black	10.6% (1327)	38.8% (473)	7.6% (854)	
** **Other race	6.5% (347)	10.4% (57)	6.1% (290)	
**Education**				.005
** **≥High school	82.6% (5329)	77.8% (650)	83.1% (4679)	
** **<High school	17.4% (1960)	22.2% (270)	16.9% (1690)	
**Marital status**				.026
** **Married/cohabiting	65.0% (4506)	59.8% (496)	65.6% (4010)	
** **Single/separated/divorced/widowed	35.0% (2786)	40.2% (423)	34.4% (2363)	
**PIR group**				<.001
** **≥1.30	80.4% (4639)	72.4% (546)	81.3% (4093)	
** **<1.30	19.6% (2021)	27.6% (295)	18.7% (1726)	
**Height (cm**)	169.10 ± 0.18	168.56 ± 0.44	169.15 ± 0.19	.187
**BMI (kg/m**^**2**^)	28.81 ± 0.13	31.48 ± 0.33	28.53 ± 0.14	<.001
**BMI category**				<.001
** **Normal/underweight	30.1% (1935)	19.4% (161)	31.2% (1774)	
** **Overweight	34.0% (2511)	27.1% (252)	34.7% (2259)	
** **Obese	35.9% (2850)	53.5% (507)	34.0% (2343)	
**FEV**_**1**_ **(% predicted**)	97.30 ± 0.31	72.54 ± 0.28	99.90 ± 0.22	<.001
**FEV**_**1**_ **/FVC ratio**	0.803 ± 0.002	0.782 ± 0.003	0.805 ± 0.002	<.001
**Smoking status**				.231
** **Never smoker	58.1% (4185)	55.0% (478)	58.4% (3707)	
** **Ever smoker (former/current)	41.9% (3108)	45.0% (442)	41.6% (2666)	
**Alcohol consumption**				<.001
** **Drinker	77.8% (5074)	67.9% (574)	78.9% (4500)	
** **Nondrinker	22.2% (1834)	32.1% (300)	21.1% (1534)	
**Diabetes**				<.001
** **No	93.2% (6596)	81.9% (740)	94.4% (5856)	
** **Yes	6.8% (693)	18.1% (179)	5.6% (514)	
**Hypertension**				<.001
** **No	74.6% (5182)	59.0% (514)	76.2% (4668)	
** **Yes	25.4% (2105)	41.0% (403)	23.8% (1702)	
**C-reactive protein** (mg/dL)	0.35 ± 0.01	0.55 ± 0.05	0.33 ± 0.01	<.001
**Albumin (g/dL**)	4.29 ± 0.01	4.17 ± 0.02	4.30 ± 0.01	<.001
**Lymphocyte count** (×10^9^/L)	2.14 ± 0.01	2.25 ± 0.04	2.13 ± 0.01	.007
**CALLY**	12.63 ± 0.44	8.13 ± 0.60	13.10 ± 0.50	<.001
**ln(CALLY**)	1.71 ± 0.03	1.21 ± 0.06	1.77 ± 0.03	<.001
**ln(CALLY) quartiles**				<.001
** **Q1	22.7% (1824)	37.4% (347)	21.1% (1477)	
** **Q2	23.6% (1825)	23.6% (236)	23.6% (1589)	
** **Q3	25.6% (1823)	22.3% (204)	25.9% (1619)	
** **Q4	28.2% (1824)	16.7% (133)	29.4% (1691)	

### 3.2. Association between ln(CALLY) and PRISm

As shown in Table 2, a significant inverse association between ln(CALLY) and PRISm was observed across all models. In the fully adjusted Model 3, higher ln(CALLY) levels were associated with a significantly lower risk of PRISm in a dose-response manner (*P* for trend < .001). Compared with the lowest quartile (Q1), the adjusted odds ratios (ORs) for Q2 to Q4 were 0.71 (95% CI: 0.57–0.88), 0.63 (0.47–0.84), and 0.48 (0.35–0.66), respectively. Analyzed continuously, each standard deviation increase in ln(CALLY) was associated with a 24% reduction in PRISm risk (OR: 0.76; 95% CI: 0.68–0.85).

**Table 2:** Association between ln(CALLY) index and PRISm in multivariable logistic regression models.

Variable	Model 1 OR (95%CI)	*P*-value	Model 2 OR (95%CI)	*P*-value	Model 3 OR (95%CI)	*P*-value
ln(CALLY)	0.53(0.47,0.59)	<.001	0.54(0.48,0.61)	<.001	0.76(0.68,0.85)	<.001
ln(CALLY) quartiles						
Q1	1.00		1.00		1.00	
Q2	0.56(0.47,0.68)	<.001	0.56(0.45,0.68)	<.001	0.71(0.57,0.88)	.007
Q3	0.49(0.38,0.63)	<.001	0.49(0.36,0.66)	<.001	0.63(0.47,0.84)	.007
Q4	0.32(0.25,0.41)	<.001	0.32(0.25,0.43)	<.001	0.48(0.35,0.66)	<.001
*P* for trend		<.001		<.001		<.001

### 3.3. Dose-response relationship

The RCS analysis confirmed a monotonic, inverse linear relationship between ln(CALLY) and PRISm risk (Fig. 2). The risk of PRISm decreased steadily with increasing ln(CALLY) values (*P* for overall < .001), with no evidence of significant nonlinearity (*P* for nonlinearity = .707).

**Figure 2.**
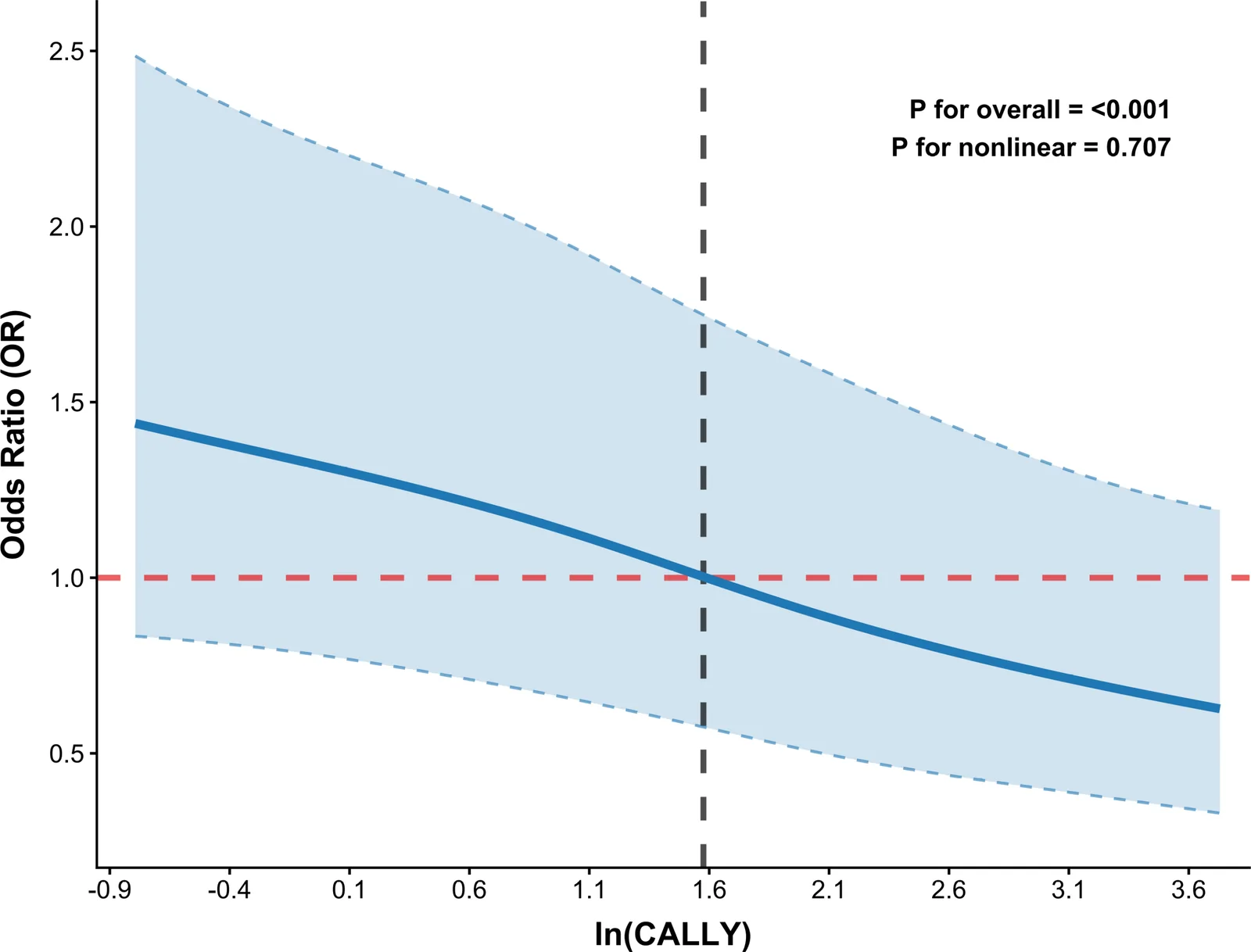
Dose-response relationship between ln(CALLY) index and the odds of preserved ratio impaired spirometry (PRISm). The solid blue line is the adjusted odds ratio (OR) from restricted cubic spline analysis in the fully adjusted Model 3. The reference point (OR = 1) is the median ln(CALLY) value (1.58, dashed black vertical line). The dashed red horizontal line is the null effect, and the shaded area is the 95% confidence interval. *P* for overall < .001 and *P* for nonlinearity = .707, confirming a monotonic inverse linear association.

The solid blue line represents the adjusted odds ratio (OR) derived from restricted cubic spline analysis within the fully adjusted model (Model 3). The reference point (OR = 1) is set at the median ln(CALLY) value (1.58), indicated by the dashed black vertical line. The dashed red horizontal line denotes the null effect (OR = 1). The shaded area represents the 95% confidence interval. *P* for overall < .001; *P* for nonlinear = .707.

### 3.4. Subgroup and interaction analyses

The inverse association was consistent across most prespecified subgroups (Table 3). A statistically significant interaction was observed with sex (*P* for interaction = .021), suggesting a potentially stronger protective association in women (OR for Q4 vs Q1: 0.39; 95% CI: 0.27–0.58) than in men (OR: 0.64; 95% CI: 0.39–1.06). A significant interaction was also detected for race/ethnicity (*P* for interaction = .036).

**Table 3:** Subgroup analysis and interaction test results of the association between ln(CALLY) index and PRISm.

Characteristic	OR (95% CI)	*P*-value	*P*-value for interaction
**Age group**			.580
** **< 60 yr	0.47(0.32,0.71)	.002	
** **≥ 60 yr	0.51(0.32,0.83)	.015	
**Gender**			.021
** **Female	0.39(0.27,0.58)	<.001	
** **Male	0.64(0.39,1.06)	.104	
**Race**			.036
** **Non-hispanic White	0.41(0.24,0.69)	.003	
** **Mexican American	0.29(0.05,1.58)	.170	
** **Other hispanic	1.09(0.45,2.68)	.850	
** **Non-hispanic Black	0.56(0.35,0.89)	.028	
** **Other race	0.82(0.14,4.82)	.829	
**Education**			.581
** **≥ High school	0.48(0.33,0.70)	.002	
** **< High school	0.44(0.27,0.74)	.007	
**Marital status**			.487
** **Married/cohabiting	0.48(0.30,0.76)	.007	
** **Single/separated/divorced/widowed	0.47(0.27,0.82)	.017	
**PIR group**			.597
** **≥ 1.30	0.47(0.31,0.72)	.003	
** **< 1.30	0.52(0.33,0.82)	.012	
**BMI category**			.166
** **Normal/underweight	0.30(0.13,0.72)	.017	
** **Overweight	0.84(0.44,1.62)	.608	
** **Obese	0.47(0.27,0.82)	.017	
**Smoking status**			.893
** **Never smoker	0.44(0.30,0.65)	<.001	
** **Ever smoker (former/current)	0.51(0.32,0.84)	.016	
**Alcohol consumption**			.485
** **Drinker	0.55(0.34,0.91)	.034	
Nondrinker	0.46(0.31,0.67)	.001	
** Diabetes**			.205
** **No	0.43(0.29,0.63)	<.001	
** **Yes	0.98(0.32,2.98)	.966	
**Hypertension**			.141
** **No	0.35(0.23,0.55)	<.001	
** **Yes	0.76(0.45,1.29)	.329	

### 3.5. Sensitivity analyses

The primary findings were robust in sensitivity analyses. Treating BMI as a continuous variable yielded nearly identical effect estimates (Table S1, Supplemental Digital Content 1). Results from multiple imputation for missing covariates were also consistent with the complete-case analysis (Table S2, Supplemental Digital Content 2).In the sensitivity analysis using GLI-2012 z-scores, the inverse association between ln(CALLY) and PRISm remained robust. Compared with Q1, the fully adjusted OR for Q4 was 0.52 (95% CI: 0.41–0.66, *P* < .001), and each 1-SD increase in ln(CALLY) was associated with an OR of 0.75 (95% CI: 0.69–0.82, *P* < .001) (Table S3, Supplemental Digital Content 3).

## 4. Discussion

This study, based on a large-scale, nationally representative sample from NHANES 2007–2010, provides the first systematic investigation into the association between the ln(CALLY) index and the risk of PRISm. After full adjustment for multiple potential confounders, we found that a higher ln(CALLY) index was significantly and inversely associated with a lower prevalence of PRISm. This index reflects a more favorable inflammatory-nutritional balance, and we observed evidence of a clear dose-response relationship. Restricted cubic spline analysis further confirmed that this association was monotonic and linear, with no significant nonlinear trend detected. Subgroup analysis suggested that this protective association might be more pronounced among females and exhibited heterogeneity across racial and ethnic groups. Sensitivity analyses confirmed the robustness of the primary findings.

PRISm, as a heterogeneous clinical phenotype, likely involves intertwined pathophysiological mechanisms related to systemic low-grade inflammation, metabolic dysregulation, and abnormal tissue repair.^[13–15]^ Our findings support the hypothesis that systemic inflammation-nutrition imbalance may play an important role in the pathogenesis of PRISm. In the CALLY index, a lower CRP level likely indicates a reduced systemic inflammatory burden, while a higher albumin level suggests better nutritional status and antioxidant capacity. Both factors may protect the lung parenchyma and airway function by mitigating oxidative stress and inflammatory damage in the pulmonary microenvironment.^[16,17]^In addition to systemic inflammation, PRISm is a heterogeneous phenotype that may be associated with obesity, restrictive physiology, suboptimal inspiration, cardiac disease, and other conditions. Our cross-sectional analysis cannot distinguish among these subtypes.

Previous research has primarily focused on the relationship between single inflammatory markers (such as CRP) or nutritional indicators (such as albumin) and lung function impairment. This study innovatively employs the composite CALLY index, which simultaneously captures information across three dimensions: inflammation, nutrition, and immunity. This may offer a more comprehensive reflection of the pathophysiological state underlying impaired lung function. The observed linear dose-response relationship in this study suggests that improving the inflammatory-nutritional balance may confer continuous benefits in reducing PRISm risk, presenting a potential target for public health interventions. More importantly, all components of the CALLY index are routinely measured in clinical practice, making it a potentially cost-effective and easily implementable tool for risk assessment compared to novel or specialized biomarkers.

The finding of a significant gender interaction warrants an in-depth discussion. The stronger protective association observed in females may be related to factors such as hormonal levels, body fat distribution, and sex-based differences in immune responses.^[18]^ This interaction suggests that the pathophysiological pathways linking systemic inflammation-nutrition status to lung function may differ by sex, warranting further investigation into sex-specific mechanisms. Future mechanistic studies are needed to further elucidate the biological basis of this gender difference. The observed heterogeneity across racial and ethnic groups suggests that genetic background, social environment, and lifestyle factors may modify the association between the CALLY index and PRISm, highlighting the necessity for precision prevention strategies. The heterogeneity underscores the importance of considering social determinants of health and genetic diversity in both research and future clinical applications.

Strengths of this study include the use of high-quality, nationally representative NHANES data, rigorous statistical adjustment, and multiple sensitivity analyses to ensure the reliability of the results, as well as being the first to apply the CALLY index in respiratory disease research. Furthermore, our analysis accounted for a comprehensive set of potential confounders, including key socioeconomic factors, strengthening the robustness of the observed independent association. However, several limitations should be acknowledged. First, the cross-sectional design precludes establishing causality. Second, the PRISm diagnosis relied on a single spirometry measurement, which is susceptible to measurement error. Third, despite adjusting for a wide range of confounders, residual confounding from unmeasured factors, such as environmental exposures and genetic factors, may still exist. Finally, as NHANES data primarily represent the U.S. population, caution is needed when extrapolating the conclusions to other populations.The fixed FEV1/FVC threshold of 0.70 may misclassify airflow obstruction, especially at the extremes of age. In a sensitivity analysis using GLI-2012 z-scores, the inverse association between ln(CALLY) and PRISm persisted, suggesting that our findings are not driven by the choice of spirometric definition.

Future research directions should include validating the present findings in prospective cohorts; exploring the association between the CALLY index and PRISm imaging phenotypes, including CT-defined emphysema and airway abnormalities; and conducting interventional studies to evaluate whether interventions aimed at improving nutrition or reducing inflammation, thereby elevating the CALLY index, can prevent or delay the progression of PRISm.

## 5. Conclusion

This study demonstrates that among U.S. adults, a higher ln(CALLY) index is independently associated with a lower prevalence of PRISm, exhibiting a linear dose-response relationship. The index reflects a more favorable systemic balance between inflammation and nutrition. As an easily accessible composite biomarker derived from routine blood tests, the CALLY index may aid in identifying individuals with PRISm.Future prospective studies are needed to validate these findings and to explore whether improving inflammatory-nutritional balance can prevent or delay the progression of PRISm.

## Acknowledgments

Our gratitude goes to the NHANES study participants and the staff who worked on data collection.

## Author contributions

**Writing – original draft:** Yucai Ye.

**Writing – review & editing:** Youhong Zhao, Yanjiong Wang.

**Resources:** Liya Jin, Juanxue Zhang.

**Formal analysis:** Qiong Jia, Wenjuan Bai.






