# Supplementary material for: The CALLY index, a novel composite biomarker, is inversely associated with preserved ratio impaired spirometry (PRISm): A nationally representative cross-sectional study
Source: Medicine (Baltimore). 2026 Nov 6;105(45):e51472. doi: 10.1097/MD.0000000000051472 (PMC13747420; doi:10.1097/MD.0000000000051472)
Supplement: Supplementary file 2 [file medi-105-e51472-s002.docx]

**Supplementary Table 2 :Multiple imputation sensitivity analysis of the association between ln(CALLY) index and PRISm risk**

| **Variable** | **Model 1**  **OR (95%CI)** | **P-value** | **Model 2**  **OR (95%CI)** | **P-value** | **Model 3**  **OR (95%CI)** | **P-value** |
| --- | --- | --- | --- | --- | --- | --- |
| **ln(CALLY)** | 0.72(0.67,0.78) | <0.001 | 0.73(0.67,0.79) | <0.001 | 0.80(0.74,0.87) | <0.001 |
| **ln(CALLY) quartiles** |  |  |  |  |  |  |
| **Q1** | 1.00 |  | 1.00 |  | 1.00 |  |
| **Q2** | 0.55(0.47,0.68) | <0.001 | 0.57(0.47,0.68) | <0.001 | 0.68(0.56,0.83) | 0.007 |
| **Q3** | 0.49(0.38,0.63) | <0.001 | 0.49(0.38,0.63) | <0.001 | 0.63(0.47,0.86) | 0.007 |
| **Q4** | 0.32(0.25,0.41) | <0.001 | 0.32(0.25,0.41) | <0.001 | 0.47(0.36,0.63) | <0.001 |
| **P for trend** |  | <0.001 |  | <0.001 |  | <0.001 |

**Note:** Note: Model 1 = Unadjusted; Model 2 = Adjusted for age, sex, race/ethnicity; Model 3 = Fully adjusted model; Reference group = Q1 (lowest ln(CALLY) quartile); Trend test = *P* for trend across ln(CALLY) quartiles; Multiple imputation: m=10 (Rubin’s rules), with predictive mean matching (continuous) and polytomous regression (categorical).
