# Supplementary material for: The CALLY index, a novel composite biomarker, is inversely associated with preserved ratio impaired spirometry (PRISm): A nationally representative cross-sectional study
Source: Medicine (Baltimore). 2026 Nov 6;105(45):e51472. doi: 10.1097/MD.0000000000051472 (PMC13747420; doi:10.1097/MD.0000000000051472)
Supplement: Supplementary file 3 [file medi-105-e51472-s003.docx]

## **Supplementary Table 3：Sensitivity analysis of the association between ln(CALLY) and PRISm risk using GLI-2012 z-scores to define airflow obstruction (lower limit of normal)**

| **Variable** | **Model 1**  **OR (95%CI)** | **P-value** | **Model 2**  **OR (95%CI)** | **P-value** | **Model 3**  **OR (95%CI)** | **P-value** |
| --- | --- | --- | --- | --- | --- | --- |
| **ln(CALLY)** | 0.69(0.63,0.74) | <0.001 | 0.70(0.63,0.76) | <0.001 | 0.75(0.69,0.82) | <0.001 |
| **ln(CALLY) quartiles** |  |  |  |  |  |  |
| **Q1** | 1.00 |  | 1.00 |  | 1.00 |  |
| **Q2** | 0.63(0.53,0.74) | <0.001 | 0.64(0.53,0.77) | <0.001 | 0.72(0.60,0.87) | 0.004 |
| **Q3** | 0.53(0.42,0.66) | <0.001 | 0.53(0.42,0.68) | <0.001 | 0.62(0.49,0.77) | <0.001 |
| **Q4** | 0.38(0.31,0.47) | <0.001 | 0.41(0.32,0.52) | <0.001 | 0.52(0.41,0.66) | <0.001 |
| **P for trend** |  | <0.001 |  | <0.001 |  | <0.001 |

## **Note:**Airflow obstruction was defined as GLI-2012 FEV₁/FVC z-score < −1.645 (lower limit of normal). PRISm was defined as FEV₁/FVC z-score ≥ −1.645 and FEV₁ < 80% predicted. Model 1 = unadjusted; Model 2 = adjusted for age, sex, and race/ethnicity; Model 3 = fully adjusted for age, sex, race/ethnicity, education, marital status, poverty-income ratio, smoking status, alcohol consumption, BMI (continuous), diabetes, and hypertension. Reference group = Q1 (lowest ln(CALLY) quartile). Trend test = P for trend across ln(CALLY) quartiles. Sample size: N = 8,470; PRISm cases = 1,505.
